# Immunomodulation and Generation of Tolerogenic Dendritic Cells by Probiotic Bacteria in Patients with Inflammatory Bowel Disease

**DOI:** 10.3390/ijms21176266

**Published:** 2020-08-29

**Authors:** Shaghayegh Baradaran Ghavami, Abbas Yadegar, Hamid Asadzadeh Aghdaei, Dario Sorrentino, Maryam Farmani, Adil Shamim Mir, Masoumeh Azimirad, Hedieh Balaii, Shabnam Shahrokh, Mohammad Reza Zali

**Affiliations:** 1 Basic and Molecular Epidemiology of Gastrointestinal Disorders Research Center, Research Institute for Gastroenterology and Liver Diseases, Shahid Beheshti University of Medical Sciences, Tehran 1985717413, Iran; sh.bghavami@yahoo.com (S.B.G.); hamid.assadzadeh@gmail.com (H.A.A.); maryam_farmani4@yahoo.com (M.F.); 2 Foodborne and Waterborne Diseases Research Center, Research Institute for Gastroenterology and Liver Diseases, Shahid Beheshti University of Medical Sciences, Tehran 1985717413, Iran; a.yadegar@sbmu.ac.ir (A.Y.); rad.masy@gmail.com (M.A.); 3 IBD Center, Division of Gastroenterology, Virginia Tech Carilion School of Medicine, Roanoke, VA 24016, USA; 4 Department of Clinical and Experimental Medical Sciences, University of Udine School of Medicine, 33100 Udine, Italy; 5 Department of Internal Medicine, Roanoke Memorial Hospital, Carilion Clinic, VA 24014, USA; dr.adilshamim@gmail.com; 6 Gastroenterology and Liver Diseases Research Center, Research Institute for Gastroenterology and Liver Diseases, Shahid Beheshti University of Medical Sciences, Tehran 1985717413, Iran; hedie.baalaei@gmail.com (H.B.); shabnamshahrokh@gmail.com (S.S.); nnzali@hotmail.com (M.R.Z.)

**Keywords:** inflammatory bowel diseases, Crohn’s disease, ulcerative colitis, dendritic cells, immune tolerance, probiotic bacteria, anti-inflammatory response

## Abstract

In inflammatory bowel diseases (IBD), the therapeutic benefit and mucosal healing from specific probiotics may relate to the modulation of dendritic cells (DCs). Herein, we assessed the immunomodulatory effects of four probiotic strains including *Lactobacillus salivarius*, *Bifidobacterium bifidum*, *Bacillus coagulans* and *Bacillus subtilis* natto on the expression of co-stimulatory molecules, cytokine production and gene expression of signal-transducing receptors in DCs from IBD patients. Human monocyte-derived DCs from IBD patients and healthy controls were exposed to four probiotic strains. The expression of co-stimulatory molecules was assessed and supernatants were analyzed for anti-inflammatory cytokines. The gene expression of toll-like receptors (TLRs), IL-12p40 and integrin αvβ8 were also analyzed. CD80 and CD86 were induced by most probiotic strains in ulcerative colitis (UC) patients whereas only *B. bifidum* induced CD80 and CD86 expression in Crohn’s disease (CD) patients. IL-10 and TGF-β production was increased in a dose-independent manner while TLR expression was decreased by all probiotic bacteria except *B. bifidum* in DCs from UC patients. TLR-4 and TLR-9 expression was significantly downregulated while integrin ß8 was significantly increased in the DCs from CD patients. IL-12p40 expression was only significantly downregulated in DCs from CD patients. Our findings point to the general beneficial effects of probiotics in DC immunomodulation and indicate that probiotic bacteria favorably modulate the expression of co-stimulatory molecules, proinflammatory cytokines and TLRs in DCs from IBD patients.

## 1. Introduction

Inflammatory bowel diseases (IBD), including Crohn’s disease (CD) and ulcerative colitis (UC), are chronic, relapsing disorders thought to be multifactorial in origin and involve host immunity, genetic, microbial and environmental factors [1,2]. Ultimately, the interaction of all these factors results in an expansion of the autoreactive T cells and altered cytokine production, which then lead to severe inflammation and injury to gut epithelial cells [3,4]. Dendritic cells (DCs) are the most potent antigen-presenting cells (APCs), which can effectively induce antigen-specific immune responses by modulating both tolerance and immunity against microbial antigens [5]. Currently, several clinical trials are ongoing to explore the effectiveness of tolerogenic DCs (tol-DCs) as an alternative therapeutic option in immune-mediated diseases such CD [6]. These clinical-grade tol-DCs have a semimature phenotype that exhibits low levels of T-cell costimulatory properties, and have a reduced capacity to produce proinflammatory cytokines compared to anti-inflammatory molecules, particularly through the expansion and/or induction of regulatory T cells (Treg) [7].

Probiotics are live nonpathogenic microorganisms that, when administered in adequate amounts, confer a health benefit on the host [8]. Probiotic bacteria most frequently belong to the *Lactobacillus* and *Bifidobacterium* species which are well known to exert beneficial effects in human and animal health [9,10]. Moreover, the regular intake of other probiotic species, such as *Bacillus subtilis* and *Bacillus coagulans*, contributes to immune modulation by restoring the microbial balance [11,12]. These probiotics are currently being used to prevent or treat various clinical conditions, most commonly gastrointestinal disorders [13]. Although the overall remission rates in patients and/or significant clinical benefits in IBD patients have not been systematically proven, probiotics do provide a benefit in terms of the reduction in disease activity in mild to moderate UC [14].

The exact mechanisms by which probiotics modulate the immune system are not completely understood. Current evidence suggests that probiotics might modulate the immune system by downregulating the pathogen recognition receptors (PRR) expressed on macrophages, such as the families of toll-like receptors (TLRs) and the C-type lectins which mediate the recognition of pathogen-associated molecular patterns (PAMPs) on bacterial cells [15,16,17]. Previous studies have demonstrated that probiotics can modulate the DC-mediated cytokine secretion in a strain-specific manner [15,18,19]. However, a number of aspects related to the impact of the strain-specific induction of cytokine secretion on DC differentiation and maturation remain unclear. Therefore, we intended to compare the direct immunomodulatory effects of the listed probiotic strains on DC function and induction of a semimature phenotype or tol-DCs. We assessed the immunomodulatory efficacy of four probiotic strains, including *Lactobacillus salivarius*, *Bifidobacterium bifidum*, *Bacillus coagulans* and *Bacillus subtilis* natto, in increasing the multiplicity of infection (MOI) by 10 (10^7^ CFU/mL) and 100 (10^8^ CFU/mL) for each strain on the induction of pro- and anti-inflammatory cytokines (IL-12, IL-10 and TGF-β); co-stimulatory molecules (CD80 and CD86); signal-transducing receptors (TLR-2, TLR-4, TLR-9 and integrin αvβ8) in human monocyte-derived DCs from six IBD patients (three UC, three CD) and three healthy controls.

## 2. Results

### 2.1. DC Characterization, Induction of CD80 and CD86 and Cytokine Production

**Figure 1:**
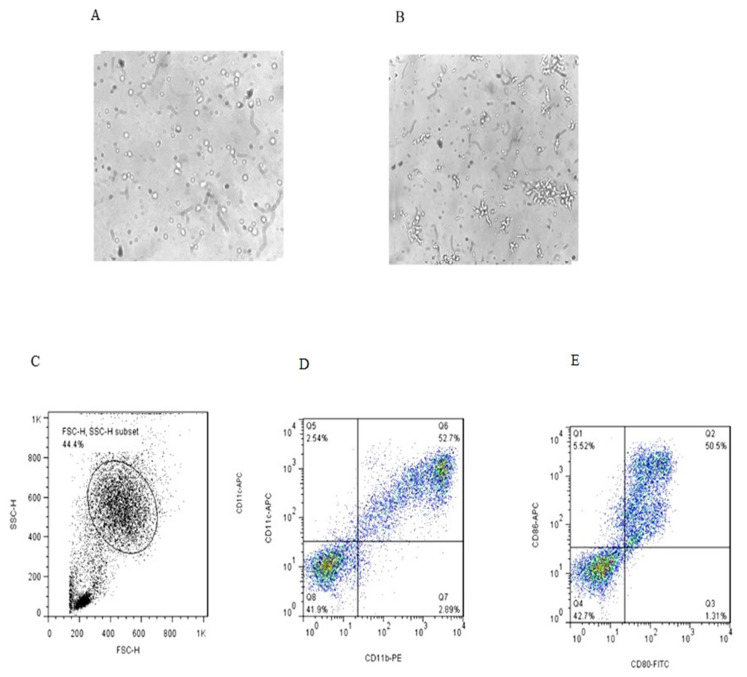
Microscopic and flow cytometric analysis of dendritic cells on the day 6 of culture (**A**,**B**) (magnification 40×); the cells in the gated population (**C**) were further analyzed for CD11C^+^/CD11b^+^ expression (**D**) and CD80/CD86 expression (**E**).

Morphological and phenotypic differences of monocytes and immature DCs (iDCs) derived from one of the healthy controls in six days of culture are shown in Figure 1 (microscopic and flow cytometric analysis). At day 6 of culture, the iDCs were analyzed for CD11b, CD11c, CD80 and CD86 markers.

**Figure 2:**
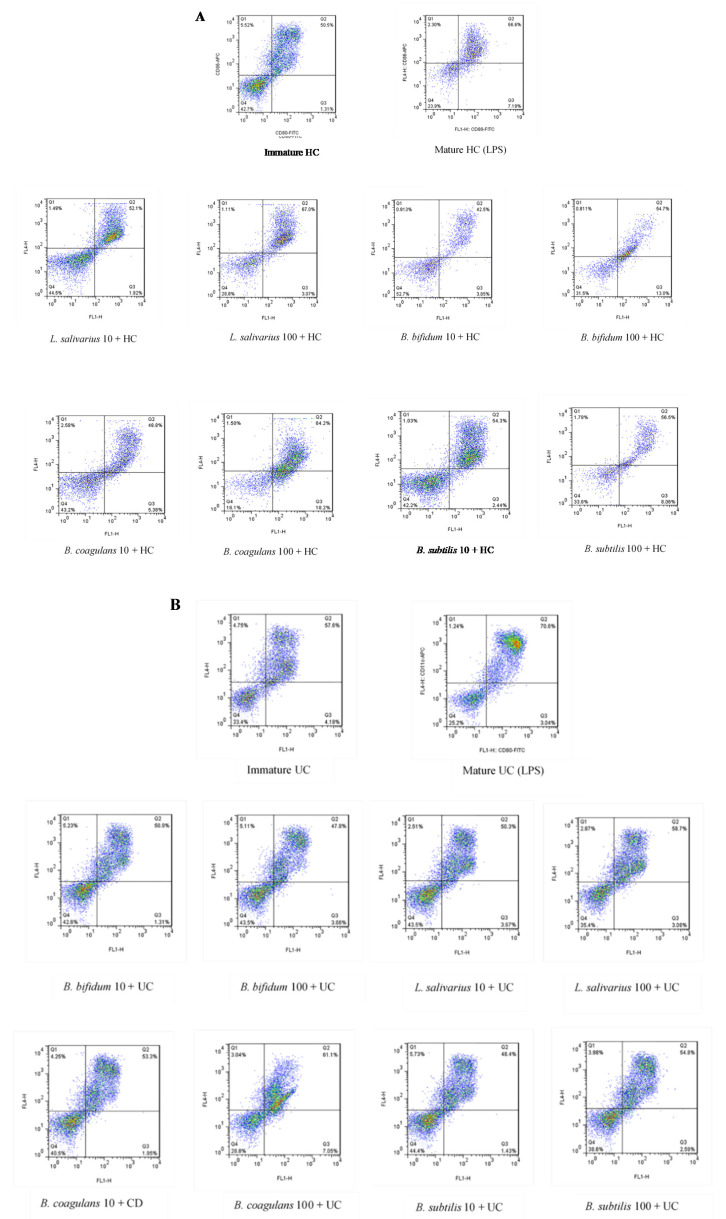

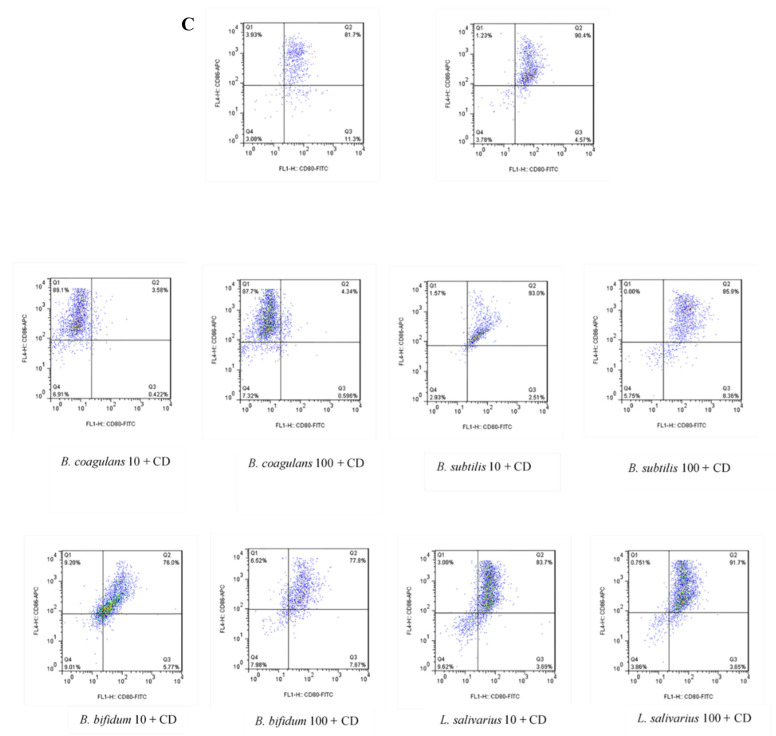
Effects of probiotics on dendritic cells’ (DCs) surface marker expression in maturation pathway after treatment for 24 h. (**A**) (HC); (**B**) (UC); (**C**) (CD). HC: healthy controls, UC: ulcerative colitis, CD: Crohn’s disease.

**Figure 3:**
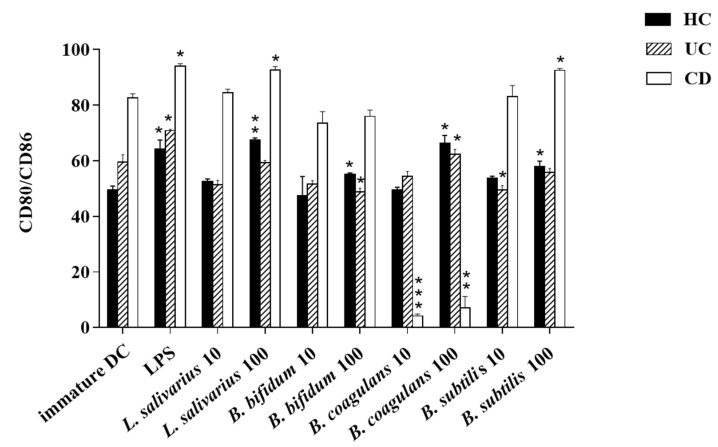
Bar graph chart representing the expression of CD80/CD86 in enriched DC population with the respective probiotic strains. HC: healthy controls, UC: ulcerative colitis, CD: Crohn’s disease. * *p* < 0.05, ** *p* < 0.01, and *** *p* < 0.001.

The upregulation of DCs surface markers (CD80 and CD86) upon stimulation with probiotic bacteria are presented in Figure 2A–C and Figure 3. In the DCs from healthy controls, *L. salivarius* and *B. coagulans* induced the expression of co-stimulatory surface molecules at MOI 10 (52.1% and 48.8%, respectively), while *B. bifidum* and *B. subtilis* natto induced these markers at both MOI 10 (42.5% and 54.3%, respectively) and 100 (54.7% and 56.5%, respectively). In the DCs from UC patients, *L. salivarius*, *B. bifidum* and *B. subtilis* natto induced these markers at both MOI 10 (50.3%, 50.9% and 48.4%, respectively) and 100 (58.7%, 47.8% and 54.8%, respectively). In the DCs from CD patients, only *B. bifidum* at both MOI 10 (76%) and 100 (77.6%) could induce them.

**Figure 4:**
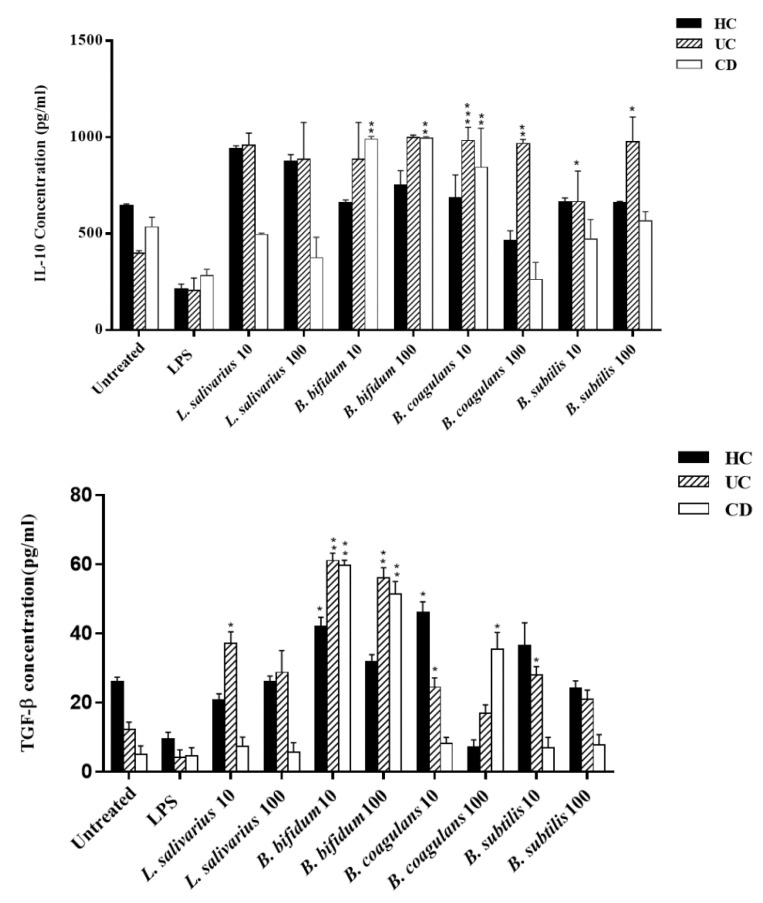
Effects of probiotics on anti-inflammatory cytokine production by DCs. HC: healthy controls, UC: ulcerative colitis, CD: Crohn’s disease. * *p* < 0.05, ** *p* < 0.01, and *** *p* < 0.001.

Figure 4 (left panel) presents the IL-10 secretion induced by the various probiotic bacteria used in this study. As shown, all four bacteria significantly induced the IL-10 production in DCs from healthy controls, and UC patients at both MOIs (*p* < 0.05), as compared to the untreated and lipopolysaccharide (LPS)-treated DCs. In the DCs from CD patients, *B. bifidum* significantly induced the IL-10 production in DCs at both MOIs and *B. coagulans* significantly induced IL-10 production at MOI 10 (*p* < 0.05).

Figure 4 (right panel) also presents the TGF-β production stimulated by the four probiotic bacteria used in this study. In general, higher levels of TGF-β were induced in UC compared to CD patients. All four probiotic bacteria induced higher levels of TGF-β than those from untreated DCs or LPS stimulated DCs in UC. In UC patients, *L. salivarius* significantly induced TGF-β production in a dose-independent manner (*p* < 0.05). *B. coagulans* induced higher levels of TGF-β in DCs from UC patients at MOI 10 (*p* < 0.05). *B. subtilis* at MOI 10 increased levels of TGF-β only in DCs from UC patients (*p* < 0.05). For CD patients, significantly higher levels of TGF-β were present in DCs treated with *B. coagulans* (*p* < 0.05) at MOI 100. In both UC and CD patients, the highest levels of induction were present in DCs treated with *B. bifidum* at both MOIs compared with untreated or LPS-treated DCs (*p* < 0.05).

### 2.2. Expression of TLRs, Integrin ß8 (ITG ß8) and IL-12p40

**Figure 5:**
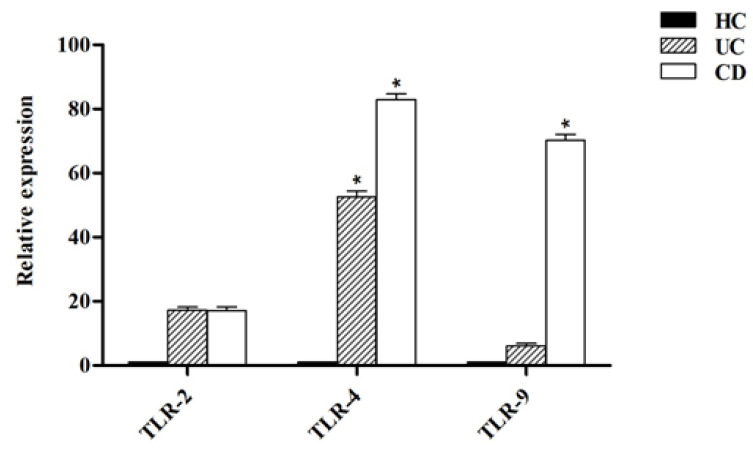
Relative expression of toll-like receptors (TLRs) in DCs from healthy controls (HC), ulcerative colitis (UC) and Crohn’s disease (CD) patients. * *p* < 0.05.

Figure 5 shows that the expression level of all analyzed TLRs was higher in DCs isolated from both UC and CD patients compared to healthy controls (*p* < 0.05). The TLR-2 expression in UC and CD patients was slightly higher compared to healthy controls. In UC patients, the TLR-4 expression was significantly higher (*p* < 0.05) in comparison with healthy controls whereas in CD patients, both the TLR-4 and TLR-9 expression were significantly higher compared to healthy controls (*p* < 0.05).

Figure 5 shows the relative expression of TLR-2, TLR-4 and TLR-9 after stimulation of DCs with probiotic bacteria. Overall, TLR-2 expression was decreased by all probiotic bacteria, except for UC patients after stimulation by *B. bifidum* at both MOI 10 and 100 (*p* < 0.05). TLR-4 expression was significantly downregulated (*p* < 0.05) in DCs isolated from CD patients following stimulation by most probiotic bacteria. TLR-4 expression was also downregulated by most probiotic bacteria in DCs isolated from UC patients—however the difference was not statistically significant (*p* >0.05). Finally, the expression of TLR-9 was significantly downregulated in the DCs isolated from CD and UC patients as compared to LPS-stimulated DCs. This down regulation was significant in CD patients (*p* < 0.05) by *L. salivarius* at MOI 10, *B. bifidum* at MOI 10 and 100, and *B. coagulans* at MOI 10, and *B. subtilis* natto at MOI 100.

**Figure 6:**
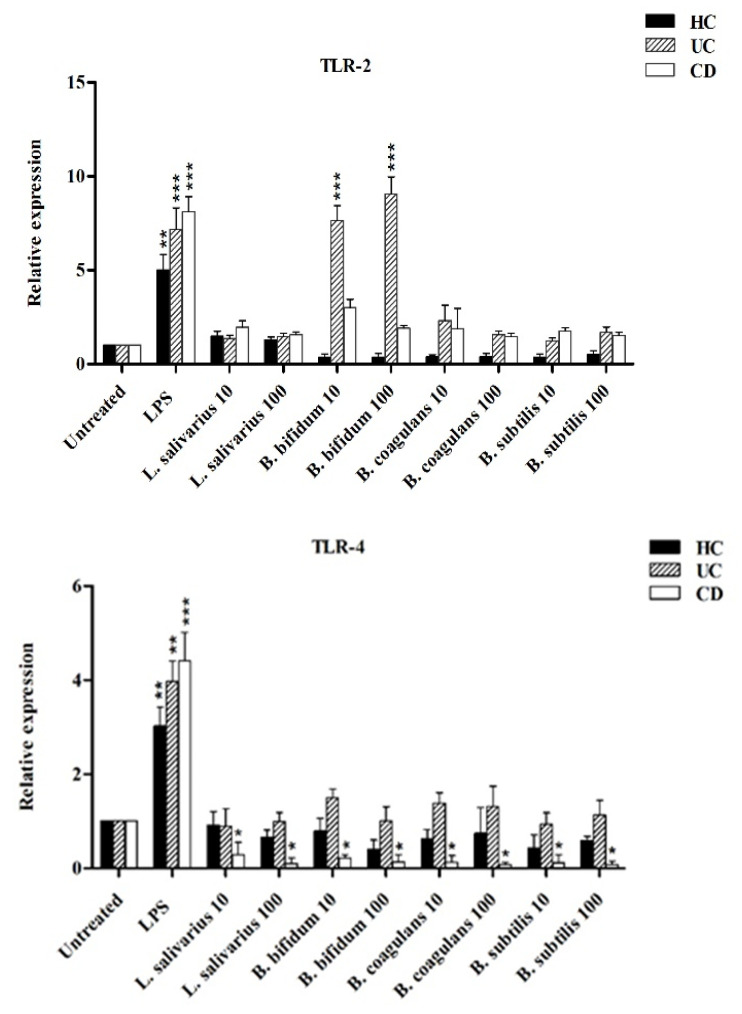

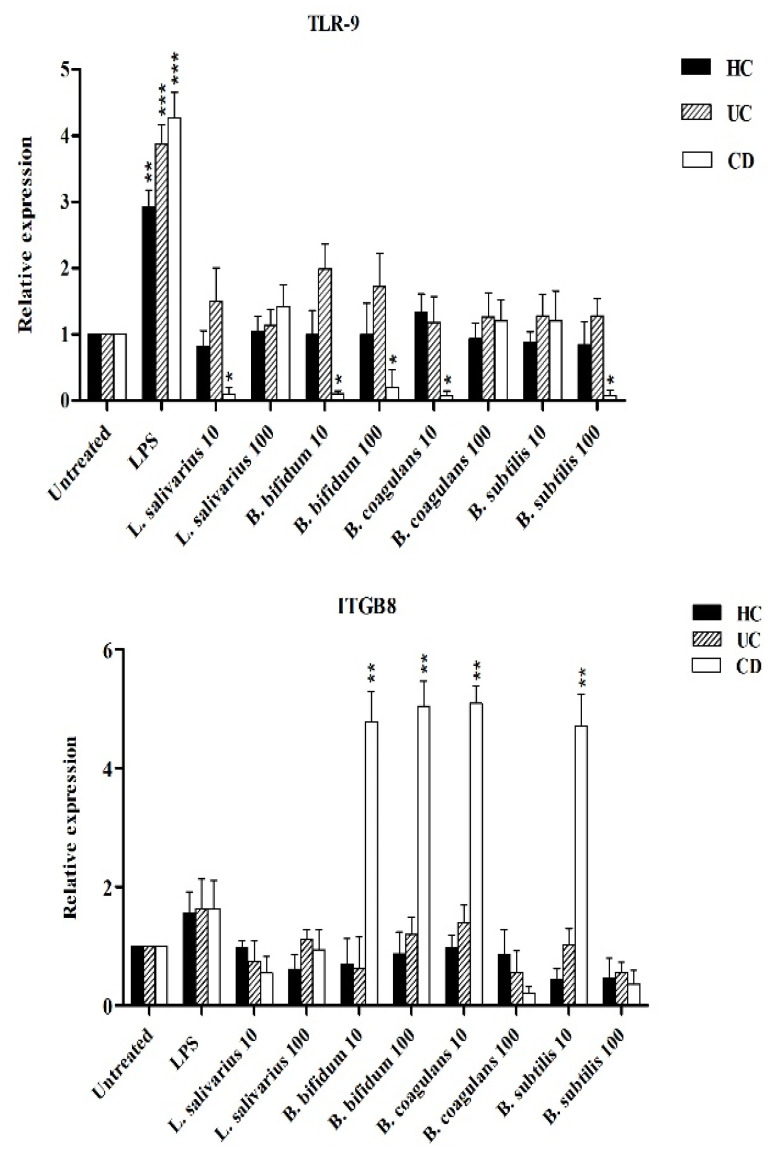
Relative expression of TLR-2, TLR-4, TLR-9 and integrin ß8 after stimulation of DCs with probiotic bacteria. * *p* < 0.05, ** *p* < 0.01, and *** *p* < 0.001.

Figure 6 shows that the expression of integrin ß8 (ITG ß8) was significantly increased by *B. bifidum* (MOI 10 and 100), *B. coagulans* (MOI 10) and *B. subtilis* natto (MOI 10) in DCs isolated from CD patients (*p* < 0.05). *L. salivarius* was unable to induce the significant expression of integrin ß8 in all DCs isolated from IBD patients and healthy controls.

**Figure 7:**
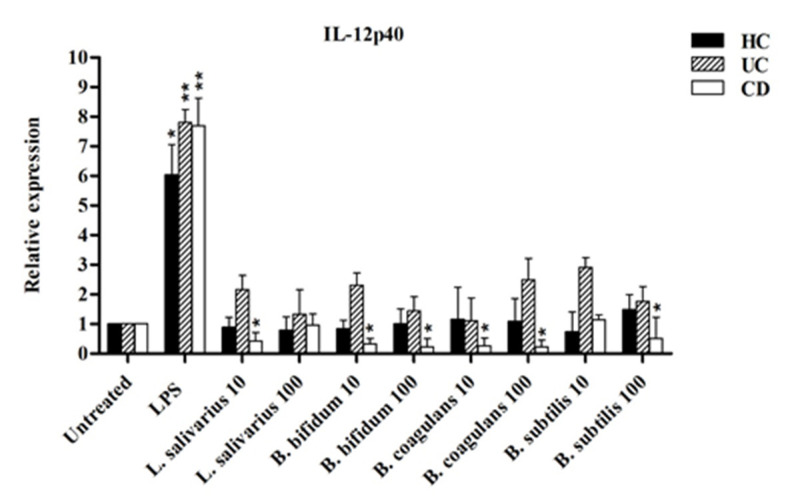
IL-12p40 expression after stimulation with probiotic bacteria. * *p* < 0.05, ** *p* < 0.01.

Figure 7 shows the relative expression of IL-12p40 after stimulation with probiotic bacteria. The expression of IL-12p40 was significantly downregulated by most probiotic bacteria in CD more than in UC patients compared to LPS-stimulated DCs. In the DCs isolated from CD patients, IL-12p40 expression was significantly decreased in a dose-independent manner (*p* < 0.05).

## 3. Discussion

Interaction between the gut microbiota and the host immune system likely plays a significant role in the pathogenesis of intestinal inflammation in IBD. A thorough understanding of these interactions is of foremost significance not only to better understand disease etiology and pathogenesis, but also for the development of targeted therapies.

Our data show increased expression of co-stimulatory molecules (CD80 and CD86) from DCs after probiotic treatment. It is well known that these markers can either inhibit or stimulate T cell responses through their interactions with cytotoxic T lymphocyte-associated antigen 4 (CTLA-4) or CD 28, respectively [20]. Although the exact mechanism remains largely unknown, it is possible that probiotic bacteria promotes CD80/86 interactions with CTLA-4, leading to the downregulation of the T cell response. This is supported by the study by Wang et al. who found that the administration of *L. reuteri* inhibits the development and progression of immune checkpoint blocker (ICB)-induced colitis (which enhances antitumor immunity by blocking CTLA-4), and that the depletion of Lactobacillus by antibiotic therapy worsens ICB colitis [21]. Our findings are also consistent with the observations of Luongo et al. who found that pre-incubation of murine DCs with lactobacilli increases the expression of the maturation markers and induces an anti-inflammatory effect [22]. However, these authors also found that bifidobacteria decreases the costimulatory surface molecule expression, which is in contrast to our results. It has been suggested that some probiotic species such as *L. reuteri* and *L. casei* influence monocyte-derived DCs through the upregulation of surface major histocompatibility complex (MHC) class II and CD86 to drive the development of Tregs leading to an elevated secretion of IL-10 [23,24]. Therefore, the induction of Tregs by probiotics, particularly through the expression of costimulatory molecules on DCs, could in theory be applied as a therapeutic approach to control inflammation in a number of immune-mediated conditions.

In our study, IL-10 and TGF-β production from stimulated DCs was significantly induced by most of the probiotic species, especially in UC patients and healthy controls (HCs). In CD patients, *B. bifidum* and *B. coagulans* showed a significant ability to induce IL-10 and TGF-β. Previous studies have shown that probiotic bacteria (*Bifidobacterium longum* and *Lactobacillus rhamnosus*) induce the release of key anti-inflammatory cytokines, including IL-10 and TGF-β, in DC culture supernatants and our results are in accordance with those studies [19,25,26]. TGF-β is a well-known negative regulator of the adaptive immune system and has been implicated in the development and function of Tregs [27]. There seems to be a noticeable variability regarding the capability of probiotic strains to stimulate the production of pro- and anti-inflammatory cytokines from DCs. Recently, data from two different studies showed that an oral/rectal administration of *Lactobacillus casei* DG and rectal infusion of *Lactobacillus reuteris* ATCC 55730 increases the expression of IL-10 in patients with mild to moderate UC [28,29]. Di Giacinto et al. also reported that administration of VSL#3 probiotic bacteria during remission can induce an immunoregulatory response through TGF-β-bearing regulatory cells and can ameliorate the severity of recurrent murine colitis [30]. Braat et al. have shown that an oral supplementation of *Lactobacillus rhamnosus* can induce in vivo peripheral T cell hyporesponsiveness, suggesting a modulation through enhanced DC-T cell interaction in cohorts of both healthy volunteers and patients with CD [31]. Thus, the induction of anti-inflammatory cytokines from DCs by probiotic bacteria likely plays a crucial role in their immunomodulatory function.

Our results showed decreased TLR-2, TLR-4 and TLR-9 expression from DCs after stimulation with probiotic bacteria. Several studies have proposed that a high expression of TLR-2 and TLR-4 may be associated with IBD pathogenesis, and ultimately can modulate the host’s susceptibility to colitis [32,33,34]. Sánchez-Muñoz et al. demonstrated that TLR-2, TLR-4 and TLR-9 expression was increased in patients with active UC, and also showed that their levels positively correlated with the inflammatory cytokines and the degree of intestinal inflammation [35]. Thus it appears that the regulation of TLR-2, TLR-4 and TLR-9 expression by probiotic bacteria likely plays a significant role in immunomodulation. TLR-2 and TLR-4 are expressed in the outer cellular membrane and primarily respond to bacterial surface-associated PAMPs, whereas TLR-9 is expressed intracellularly on the surface of endosomes and responds primarily to nucleic acid-based PAMPs from viral and bacterial agents [36]. Hoarau et al. reported that a fermentation product from *Bifidobacterium breve* C50 can induce maturation, high IL-10 production and a prolonged survival of DCs via the TLR-2 pathway [37]. Giahi et al. noted that heat-inactivated *Lactobacillus rhamnosus* GG (LGG) and *Lactobacillus delbrueckii* subsp. *bulgaricus* can downregulate TLR-4 expression [38]. Kim et al. reported that *Lactobacillus plantarum* genomic DNA (gDNA) mediates the inhibition of LPS-induced TNF-α production by suppressing TLR-2, TLR-4 and TLR-9 expression, as well as by inducing IL-1 receptor-associated kinase M (IRAK-M), which is a negative regulator of TLRs [39]. The effects and related mechanisms that involve intestinal DCs through TLR activation are not fully understood. However, it has been proposed that specific probiotic bacteria can induce Tol-DCs in a TLR-mediated pathway, and TLR activation by such microorganisms can stimulate DCs to induce the differentiation of naive T helper (Th) cells to Treg, which have an inhibitory effect on inflammatory responses produced by Th cell subsets [19,32].

Our study shows that ITG ß8 expression was significantly increased by most probiotic bacteria (except *L. salivarius*), especially in CD patients. It is well known that the β8 subunit plays a critical role alongside the αv subunit in the activation of TGF-β by DCs [40]. The expression of αv-β8 integrin is tightly regulated in DCs, and it is expressed predominantly in DCs from mesenteric lymph nodes (MLN) and intestinal lamina propria [41]. Païdassi et al. found that αv-β8 integrin expression is required for the activation of latent TGF-β and Treg generation [42]. It has also been shown that DCs lacking αv-β8 fail to induce Tregs in vitro, and mice in which myeloid cells do not express αv or their DCs do not express αv-β8 tend to develop colitis [41,43]. Hence, the expression of ITG ß8 from the specifically positioned intestinal DCs likely plays an important role in intestinal immune tolerance.

Our data also show that IL-12p40 (an important subunit of the biologically active form IL-12p70 which plays a key role in the regulation of T-cell response) was significantly downregulated by most probiotic bacteria both in CD and UC patients (in CD more than in UC). Previous studies in CD patients and animal models of colitis also suggest that intestinal inflammation is driven by the production of IL-12p40 through the TLR-mediated recognition of microbial components by DCs [44,45]. In agreement with our findings, Ng et al. demonstrated that DCs isolated from patients treated with VSL#3 probiotic mixture have a decreased TLR-2 and IL-12p40 expression (in addition to an increased IL-10 production) [17]. This mechanism is at the basis of the therapeutic effect of anti-IL-12 antibodies (ustekinumab) in CD [46].

## 4. Materials and Methods

### 4.1. Sample Collection and Probiotic Strains

In this study, 6 IBD patients (3 UC and 3 CD) with a mean age ± SD of 44 ± 2.3 and 42 ± 2.6 years respectively, and 3 healthy subjects with a mean age of 36 ± 0.57 years were enrolled. The demographics and clinical characteristics of the subjects enrolled in this study are presented in Table 1. In each case, IBD was diagnosed based on a combination of signs and symptoms, imaging, colonoscopy and pathology reports [47,48]. Clinical and demographic features were recorded for all subjects. Human peripheral blood mononuclear cells (PBMCs) were obtained from the heparinised blood of patients and healthy controls. Exclusion criteria included the recent use of biological medications (anti-TNF agents), any immune-mediated diseases (such as allergic diseases, rheumatoid arthritis, multiple sclerosis), malignancy, symptoms of acute or recent infection, and use of antibiotics within the last 4 weeks. None of the subjects were on any probiotic, prebiotic, synbiotic, hormonal, vitamin D or herbal supplements. Informed written consent was obtained from all the study participants. The study protocol was approved in November 2018 by the Ethical Review Committee of the Research Institute for Gastroenterology and Liver Diseases at Shahid Beheshti University of Medical Sciences (Project No. IR.SBMU.RIGLD.REC.1396.168).

Frozen stock strains of probiotic bacteria including *L. salivarius* ATCC 11741 (IBRC-M 10865) and *B. bifidum* PTCC 1644 were supplied from the Iranian Biological Resource Center (IBRC, Tehran, Iran), and the Iranian Research Organization for Science and Technology (IROST, Tehran, Iran), respectively. *B. coagulans* and *B. subtilis* natto were also supplied from Natures Only (Villa Park, Inc., Villa Park, CA, USA) and World Intellectual Resource Co. (Taiwan), respectively. After defrosting, *L. salivarius* and *B. bifidum* were cultured on Man–Rogosa–Sharpe (MRS) agar (Merck, Darmstadt, Germany) under anaerobic conditions (10% CO_2_, 5% H_2_, and 85% N_2_) generated by an Anoxomat^®^ Gas Exchange System (Mart Microbiology BV, Drachten, Holland) and incubated at 37 °C for 48–72 h. For *B. bifidum*, 0.05% l-cysteine hydrochloride (Sigma, Darmstadt, Germany) was added to the MRS medium. *Bacillus* strains were grown on brain heart infusion (BHI) agar (Merck, Darmstadt, Germany) at 37 °C under aerobic conditions for 24 h. All bacterial species were harvested in the exponential phase by centrifugation (5000× *g*, 5 min) and transferred to centrifuge tubes. After washing twice at 400× *g* for 10 min, the bacteria were resuspended in 1 mL phosphate buffered saline (PBS) (Gibco, Gaithersburg, MD, USA) prior to use in coinfection assay.

### 4.2. Generation and Stimulation of DCs

Each blood sample was diluted into an equal volume of PBS. PBMCs were isolated by density gradient centrifugation using Histopaque solution (Sigma-Aldrich, Darmstadt, Germany), and then DCs were derived as previously described by Bie Y et al. [49]. Briefly, the PBMCs were seeded into a T175 culture flask containing RPMI-1640 (Roswel Park Memorial Institute-1640) medium and 10% fetal bovine serum (FBS). After 2 h incubation at 37 °C in a humidified atmosphere containing 5% CO_2_, the nonadherent cells were gently removed. The adherence-isolated monocytes were further cultured for 6 days in RPMI-1640 medium containing 10% FBS, penicillin (100 U/mL), streptomycin (100 μg/mL), l-glutamine (2 mM) and supplemented with recombinant human granulocyte–macrophage colony-stimulating factor (GM-CSF) (1000 U/mL) and IL-4 (500 U/mL) (Peprotech, London, UK) as differentiation factors. We used the Trypan Blue viability test after six days of culture. We isolated DCs from the flask and mixed them with the dye and then visually examined the cells for dye uptake. Following 6 days of culture, the immature DCs were harvested and used for characterization assays and coinfection experiments.

Immature DCs were resuspended at a density of 5 × 10^5^ cells/mL in fresh RPMI-1640 medium and seeded in 24-well tissue culture plates at a final volume of 500 μL/well. Prior to coinfection, the media were replaced with antibiotic-free medium. The DCs were infected with each probiotic bacteria at MOI 1:10 and 1:100 (DCs: bacteria), and incubated for 24 h at 37 °C in a humidified 5% CO_2_ incubator. Lipopolysaccharide (LPS) (from *Escherichia coli* serotype O111:B4, Sigma-Aldrich, Darmstadt, Germany) was used as positive control at a final concentration of 2 μg/mL.

### 4.3. Cytokine Measurement, Quantitative RT-PCR and Statistical Analysis

**Figure 8:**
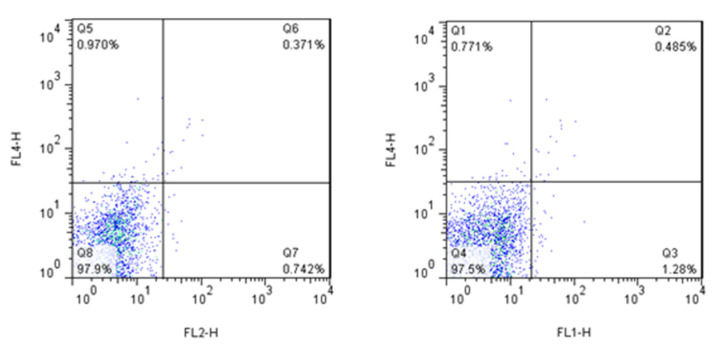
Dot-plots of cells stained with control isotype antibodies.

In order to characterize and compare the phenotype of DC populations, flow cytometry was performed on immature and coinfected DCs. Isotype-matched controls were used for all treatments. For CD86 we used mouse IgG2b and for CD80 we used mouse IgG1 from Biolegend as the Isotype control (Figure 8). The following antibodies were used for staining: phycoerythrin (PE)- or fluorescein isothiocyanate (FITC)-conjugated antibodies (BioLegend, San Diego, CA, USA) against CD11b, CD11c, CD80 and CD86. Nonspecific binding was evaluated by matched isotype controls. The DCs were analyzed using a BD FACS (Becton-Dickinson fluorescence-activated cell sorting) array flow cytometer based on counting 10,000 cells by using BD FACSDiva, Version 6. The shown graphics were performed with FlowJo Software, Version 7 (Ashland, OR, USA).

After exposure of immature DCs to various stimuli for 24 h, culture supernatants were harvested for the removal of cells and debris and stored at −70 °C until use. The production of IL-10 (Peprotech, London, UK) and TGF-β (Biolegend, San Diego, CA, USA) was quantified using ELISA kits according to the manufacturer’s guidelines. Total RNA was extracted from DCs using RNeasy Mini Kit (Qiagen, Hilden, Germany) according to the manufacturer’s protocol. The purified RNAs were quantified by using a NanoDrop^®^ ND-1000 spectrophotometer (Thermo Scientific, Waltham, MA, USA), and the RNA integrity was verified by electrophoresis on 1% (weight/volume) agarose gel.

The mRNA expression level of TLR-2, TLR-4, TLR-9, integrin αvβ8 and IL-12 were examined by quantitative RT-PCR using SYBR Green chemistry. Briefly, the extracted RNAs were transcribed into cDNA using a RevertAid RT Kit (Thermo Fisher Scientific, Inc., Waltham, MA, USA) according to the manufacturer’s protocols. Polymerase chain reaction (PCR) amplifications were carried out in a Rotor-Gene^®^ Q (Qiagen, Germany) real-time PCR system by RealQ Plus 2x Master Mix Green (Ampliqon, Odense, Denmark) using specific primers presented in Supplementary Table S1. The 10-μL PCR reactions included 0.5 μL cDNA, 5 μL SYBR^®^ Green Master Mix, 10 nM of each primer plus nuclease-free H_2_O. The PCR program was as follows: 95 °C for 15 min, followed by 40 cycles at 95 °C for 20 s and 60 °C for 60 s. A melting curve was run at the end to ensure that there was only one peak and only one product for each primer pair. All reactions were run in duplicate. The RNA input was normalized against the beta-2 microglobulin housekeeping gene. The relative gene expression levels were calculated using the delta-delta-*C*_t_ (∆∆*C*_t_) method and presented as fold change relative to the control samples.

### 4.4. Statistical Analysis

The statistical analysis was performed using GraphPad Prism software version 5.04 (GraphPad software, San Diego, CA, USA). A Student’s *t* test was applied to analyze the differences between groups. The error bars represent standard deviations (SD). *p* < 0.05 was considered statistically significant.

## 5. Conclusions

We have presented the immunomodulatory effects of specific probiotic strains on DC function, providing evidence that probiotic bacteria can influence the expression of co-stimulatory molecules and anti-inflammatory cytokines in this important subset of immune regulatory cells. One of the major limitations of our study is the small number of IBD patients involved. Furthermore, survival and maintenance of the tolerogenic status of DCs would best be evaluated in the natural intestinal microenvironment. Additionally, based on our results, the clinical effectiveness of different probiotics is likely to differ among different patients and further studies are needed to evaluate the individual patient response. Taken together, our findings underscore the potential clinical applications of probiotic bacteria in the development of new DC-based therapeutic approaches in IBD.

## Figures and Tables

**Table 1:** Demographics and clinical characteristics of study subjects.

Clinical Features	UC	CD	HC
Gender			
Females		2	2
Males	3	1	1
Age (years)	44 ± 2.3	42 ± 2.6	36 ± 0.57
BMI (kg/m^2^)	25 ± 2.4	19 ± 2.2	25 ± 0.86
Disease duration (years)	6.4 ± 4.3	7.8 ± 5.2	-
Family history	No	No	-
Intestinal surgery history	No	No	-
Smoking history	No	No	-
Phase of diseases	Flare up (Severe)	Flare up (Severe)	
Medication use			
Aminosalicylate	Yes	Yes	-
Immunomodulators	Yes	Yes	-
Anti-TNF agents	No	No	-

UC: ulcerative colitis; CD: Crohn’s disease; HC: healthy controls; BMI: body mass index.

## Supplementary Materials

The following are available online at https://www.mdpi.com/1422-0067/21/17/6266/s1, Table S1. Oligonucleotide primers used in the study.
