# Supplementary material for: Immunomodulation and Generation of Tolerogenic Dendritic Cells by Probiotic Bacteria in Patients with Inflammatory Bowel Disease
Source: Int J Mol Sci. 2020 Aug 29;21(17):6266. doi: 10.3390/ijms21176266 (PMC7503552; doi:10.3390/ijms21176266)
Supplement: Supplementary file 1 [file ijms-21-06266-s001.pdf]

Table S1. Oligonucleotide primers used in the study.

Target Genes	Primers	Nucleotide Sequence (5′–3′)	Amplicon Size (bp)	Reference
TLR2	hTLR2-F<br>hTLR2-R	TTATCCAGCACACGAATACACAG<br>AGGCATCTGGTAGAGTCATCAA	160	[1]
TLR4	hTLR4-F<br>hTLR4-R	AGACCTGTCCCTGAACCCTAT<br>CGATGGACTTCTAAACCAGCCA	147	[2]
TLR5	hTLR5-F<br>hTLR5-R	TCCCTGAACTCACGAGTCTTT<br>GGTTGTCAAGTCCGTAAAATGC	109	[3]
TLR9	hTLR9-F<br>hTLR9-R	CTGCCACATGACCATCGAG<br>GGACAGGGATATGAGGGATTTGG	121	[4]
ITGB8	ITGB8-F<br>ITGB8-R	GTGAAAGTCATATCGGATGGCG<br>GCTATCAAGAGCGAGATGAGACG	86	This study
IL-12P40	IL-12 P40-F<br>IL-12 P40-R	CATTGAGGTCATGGTGGATGC<br>TGGGTCAGGTTTGATGATGTCC	91	This study
B2M	B2 microglobulin-F<br>B2 microglobulin-R	TGCTGTCTCCATGTTTGATGTATCT<br>TCTCTGCTCCCCACCTCTAAGT	86	[5]

References

1. Wang, S.; Liu, K.; Seneviratne, C.J.; Li, X.; Cheung, G.S.P.; Jin, L.; Chu, C.H.; Zhang, C. Lipoteichoic acid from an Enterococcus faecalis clinical strain promotes TNF-α expression through the NF-κB and p38 MAPK signaling pathways in differentiated THP-1 macrophages. *Biomed. Rep.* **2015**, *3*, 697–702, doi:10.3892/br.2015.495.
2. Zhao, L.; Ma, R.; Zhang, L.; Yuan, X.; Wu, J.; He, L.; Liu, G.; Du, R. Inhibition of HIF-1a-mediated TLR4 activation decreases apoptosis and promotes angiogenesis of placental microvascular endothelial cells during severe pre-eclampsia pathogenesis. *Placenta* **2019**, *83*, 8–16, doi:10.1016/j.placenta.2019.06.375.
3. Gu, B.-X.; Wang, X.; Yin, B.-L.; Guo, H.-B.; Zhang, H.-L.; Zhang, S.-D.; Zhang, C.-L. Abnormal expression of TLRs may play a role in lower embryo quality of women with polycystic ovary syndrome. *Syst. Biol. Reprod. Med.* **2016**, *62*, 1–6, doi:10.1080/19396368.2016.1187683.
4. Yu, J.E.; Zhang, L.; Radigan, L.; Sánchez-Ramón, S.; Cunningham-Rundles, C. TLR-Mediated B Cell Defects and IFN-α in Common Variable Immunodeficiency. *J. Clin. Immunol.* **2011**, *32*, 50–60, doi:10.1007/s10875-011-9602-y.
5. Yan, D.; Yi, S.; Chiu, W.C.; Qin, L.G.; Kin, W.H.; Hung, C.T.K.; Linxiao, H.; Wai, C.K.; Yi, S.; Tao, Y.; et al. Integrated analysis of chromosome copy number variation and gene expression in cervical carcinoma. *Oncotarget* **2017**, *8*, 108912–108922, doi:10.18632/oncotarget. 22403.
